# Starch Paper-Based Triboelectric Nanogenerator for Human Perspiration Sensing

**DOI:** 10.1186/s11671-018-2786-9

**Published:** 2018-11-16

**Authors:** Zhiyuan Zhu, Kequan Xia, Zhiwei Xu, Haijun Lou, Hongze Zhang

**Affiliations:** 1 Ocean College, Zhejiang University, Zhoushan, Zhejiang 316021 People’s Republic of China; 2 Institute of Advanced Technology, Zhejiang University, Hangzhou, Zhejiang 310027 People’s Republic of China; 3 Nanjing Electronic Devices Institute, Nanjing, Jiangsu 210016 People’s Republic of China

**Keywords:** Triboelectric nanogenerator (TENG), Self-powered, Starch, Sensor

## Abstract

A disposable and ecofriendly starch paper was used to fabricate a triboelectric nanogenerator (TENG) for the sensing of human perspiration. Using cost-effective and commercially accessible materials, the starch paper-based TENG (S-TENG) can be achieved through a rapid and simple fabrication method. The output performance varies with the absorbed water content, which can be utilized for human perspiration sensing. The starch structure can be broken down in water within 4 min. The proposed S-TENGs have a considerable potential in the field of green wearable electronics.

## Introduction

The attractive attributes of flexible electronics, for example, their stretchable/bendable mechanical flexibility, small volume, and biodegradability, are expected to play a key role in disposable usage associated with electronic safety, bio-sensors, smart packing, and business cards [1–3]. In fact, flexible electronics using disposable substrates have attracted considerable attention, owing to their biocompatibility, chemical dissolvability, and environmental friendliness. Various flexible and disposable devices have therefore been used for fabricating wearable electronics [4–6], including self-power dynamic devices and intelligent sensors. In general, an additional power source is required for operating these types of wearable electronics. Nonetheless, traditional (i.e., non-portable, non-biocompatible, and non-sustainable) battery series need a constant supply of chemical power. The development of a suitable power supply is therefore essential for overcoming the challenges associated with wearable electronic gadgets.

The triboelectric nanogenerator (TENG) has been extensively investigated in the field of energy harvesting [7–12]. A TENG can convert mechanical energy derived from the environment into electrical energy and represents a novel power source, based on the processes of contact electrification and the induction of an electrostatic field [13–17]. Appropriate patterns of these devices have been extensively employed for supplying power to wearable electronic gadgets [18–21]. Furthermore, by combining TENGs with different types of triboelectric supplies, a self-powered sensor for various applications could be obtained [22–25]. However, most of the traditional TENGs are based on eco-unfriendly materials, for example, polymers that are difficult to decompose. Therefore, these TENGs may only be used on a limited basis in future applications.

Starch is a promising feedstock for developing decomposable substrates, as it is less expensive than the other alternatives, found in abundance, and renewable. Here, we have illustrated disposable TENG devices based on ecofriendly biodegradable starch papers. The materials employed are all cost-effective and commercially available. The starch paper-based TENG (S-TENG) can be constructed through a simple process where the starch paper is assembled with a metal wire. The constructed TENG can be employed as a self-powered human perspiration sensor. Furthermore, the proposed TENG holds potential for application in the area of wearable electronics.

## Method

### Assembly of the S-TENG

**Fig. 1:**
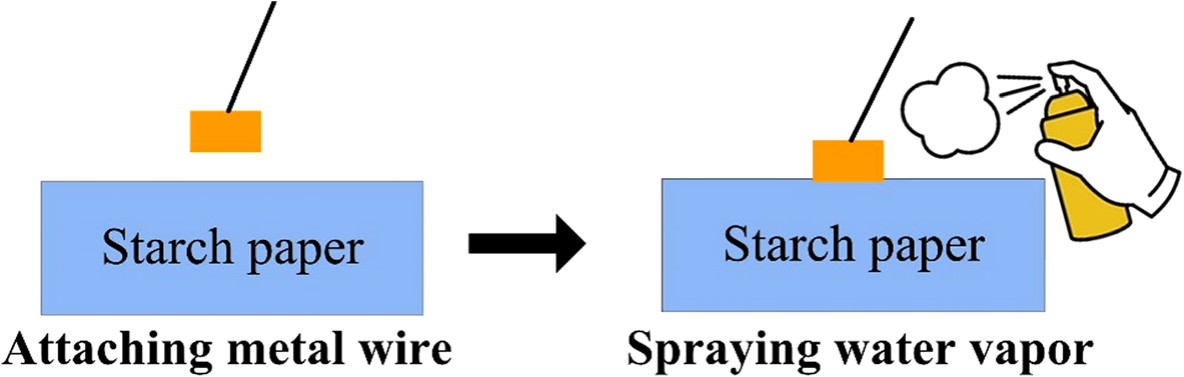
Schematic of the S-TENG assembly process

The starch paper (thickness: ~ 1 mm) was obtained from GILRO Corp. (Israel). One side of the paper is connected to a metallic wire and then sprayed with water vapors, resulting in the S-TENG. The fabrication mechanism, which is schematically demonstrated in Fig. 1, can be classified as simple and cost-effective.

### Measurements and Human Demonstration

The electronic performance was measured with a digital oscilloscope (DSOX6004A Digital Storage Oscilloscope). The fabricated S-TENG (4.4 × 4.4 cm^2^) was connected to a human elbow (metal wire facing human). In addition, the output signal of the S-TENG was measured for various durations of human biomechanical movement.

## Results and Discussion

**Fig. 2:**
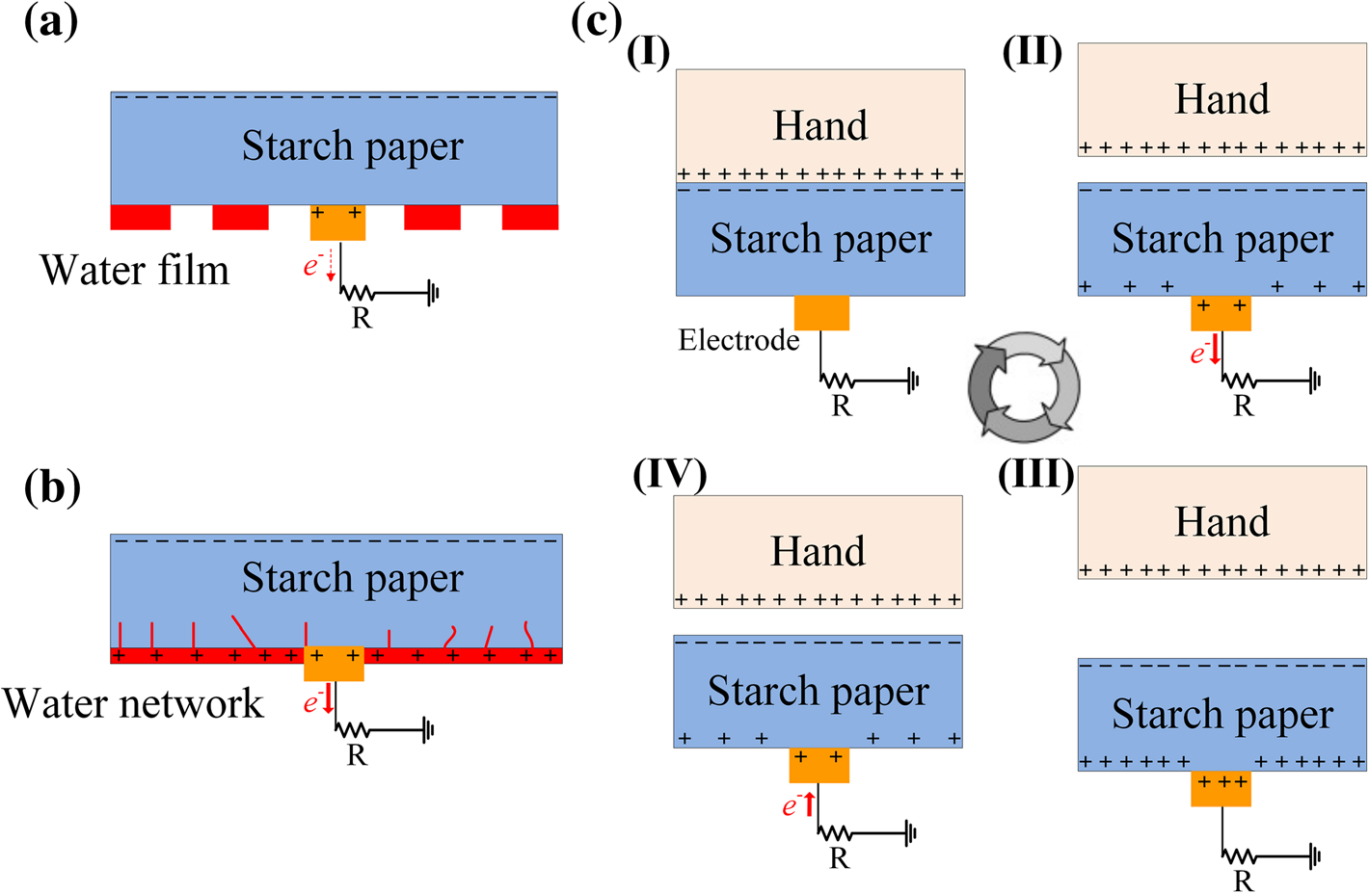
**a** A water film starts to form around the electrode side of the starch paper, **b** a water network is formed, **c** the working mechanism of the S-TENG

The working mechanism of the S-TENG is schematically shown in Fig. 2c. The proposed device is based on the coupling effect between the human hand and starch paper. When there is physical contact between the hand and the paper, the paper acquires negative charges on its surface, whereas the hand acquires positive charges. Moreover, once the hand is liberated, the overlapping area between the hand and the charged paper decreases and the charges on the paper are no longer fully balanced by those on the hand. The unstable negative charges on the starch surface force an electron flow to the ground from the back electrode of the paper. Nonetheless, when the hand approaches the paper again, the induced positive charges on the back electrode will become unstable and force the electron flow to the ground.

The results indicate that the working state of the S-TENG can be separated into two working patterns, based on the quantity of water vapor absorbed by the starch paper. The concept of charge transfer is illustrated, using the state shown in Fig. 2cII as an example. As shown in Fig. 2a, in working pattern 1, a water film is initially formed around the electrode side of the paper. Nevertheless, the charges are still partly trapped in the irregular water film, forming a potential barrier that hampers the movement of carriers. However, in working pattern 2, a water network is created (Fig. 2b), and the electronic resistance of the electrode side comprising the starch paper is greatly reduced.

**Fig. 3:**
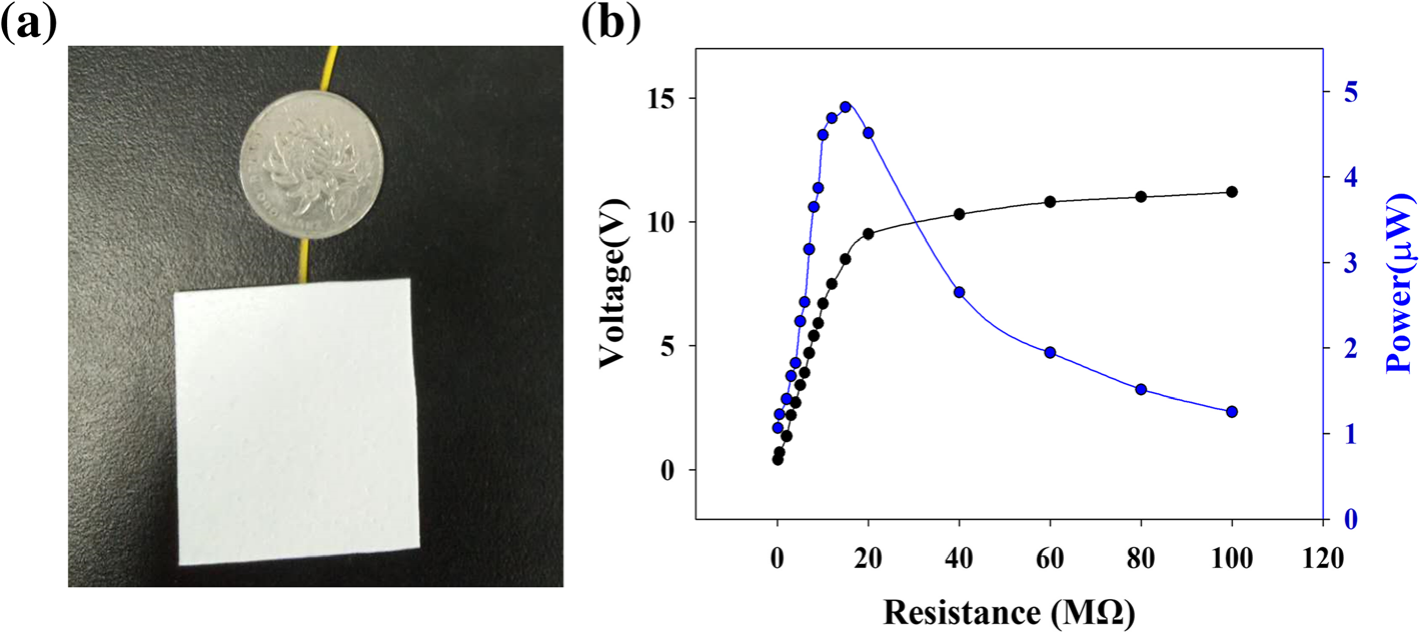
**a** Photograph and **b** electronic output of the fabricated S-TENG

**Fig. 4:**
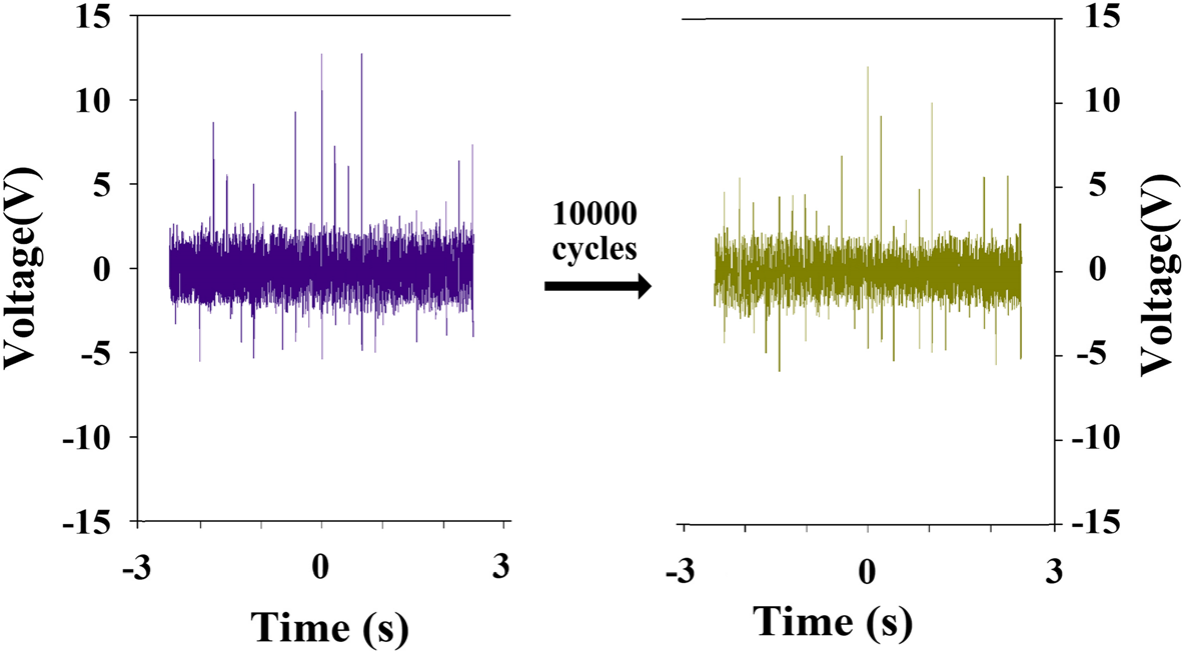
Vertical force test of fabricated S-TENG. The output voltage decreases only slightly under a loading resistance of 100 MΩ

A photograph of the fabricated S-TENG is shown in Fig. 3a. An adaptable resistor was employed as an exterior load and, for different spraying times of the water vapor, an oscilloscope was used to measure the electronic signals of the resistor. Figure 3b shows the electronic performance of the S-TENG after the first spraying. As shown in the figure, the increase in the load resistance (from 100 to 100 MΩ) yields a constant increase in the collected output voltage. However, the maximum output power is reached at a loading resistance of 15 MΩ, and hence, the internal resistance of the fabricated TENG is ~ 15 MΩ. The output voltage (i.e., 11.2 V) under a loading resistance of 100 MΩ is approximated as the open-circuit voltage, as the loading resistance is considerably greater than the approximate value of the internal resistance. The operation stability of the fabricated paper TENG was then determined. As Fig. 4 shows, the output voltage (loading resistance: 100 MΩ) of the fabricated device decreases only slightly during a vertical force test.

**Fig. 5:**
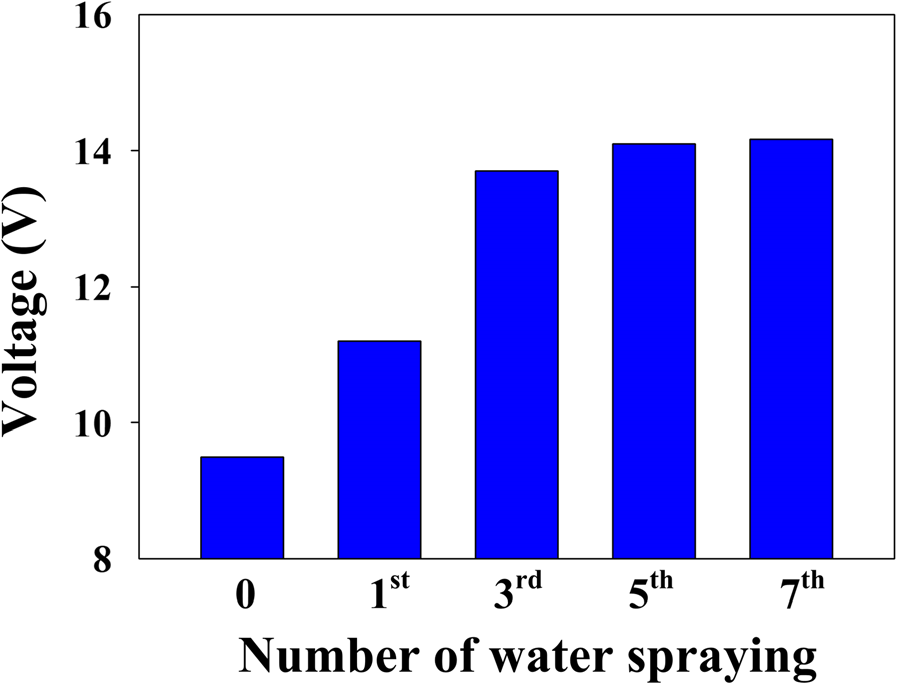
Dependence of the output voltage on the number of water spray steps

The starch paper exhibited sheet resistances of 19 MΩ, 6.1 MΩ, 1.5 MΩ, 140 KΩ, and 130 KΩ prior to water spraying and subsequent to the 1st, 3rd, 5th, and 7th spraying, respectively. The corresponding electronic activities of the S-TENG are compared, as shown in Fig. 5. The output voltage (subjected to a conforming load of 100 MΩ) increases with increased spraying times 0–3 (working pattern 1) and becomes saturated at spray times above the 3rd spraying (working pattern 2). The sensing of water-based liquids, for example, human perspiration is ensured, owing to the correlation between electronic voltage and the quantity of water vapor. This correlation can be characterized via changes in the internal resistance of the S-TENG. The reduction in the internal resistance of the S-TENG is promoted by the use of water vapor, since the introduction of water reduces the electronic resisting capacity of the starch paper. This decrease results from the formation of water conductive pathways on the surface and within the paper. Furthermore, this impact becomes particularly apparent when a film of water starts to form around the electrode side of the paper (working pattern 1). In addition, the electronic output occurring before water spraying stems mainly from the bound water of grain cells (resulting in weak electronic conduction for carriers).

**Fig. 6:**
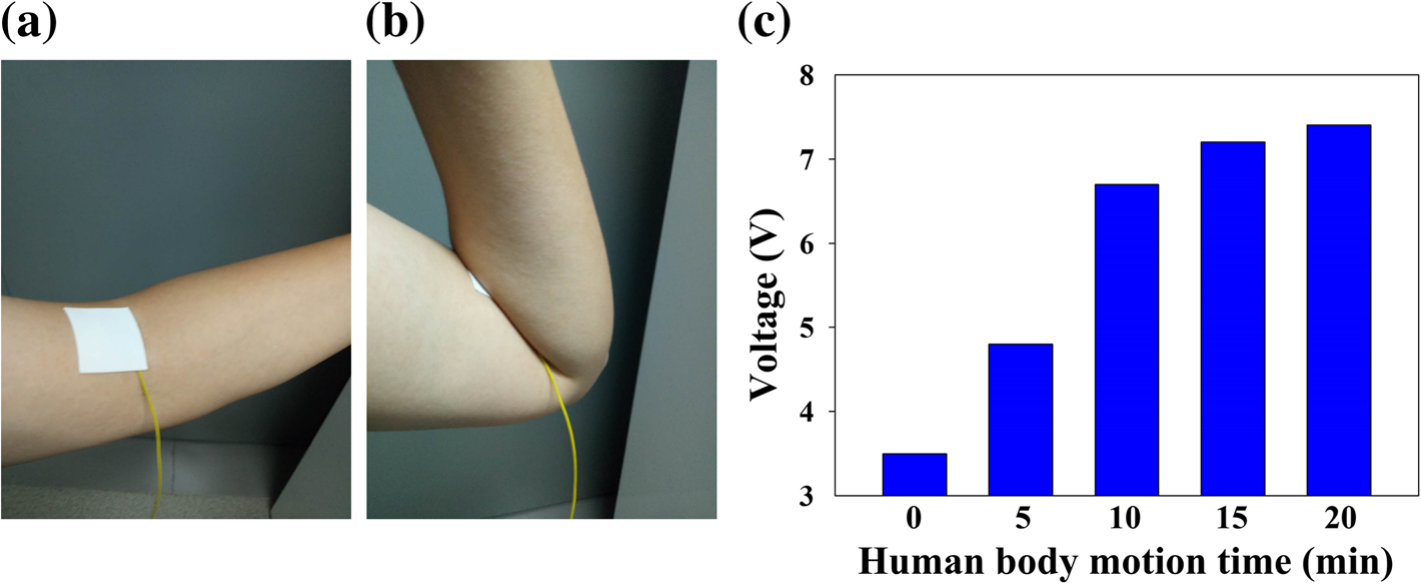
The **a**, **b** working pattern for harvesting human elbow motion energy, **c** electronic output (subject to a corresponding load of 100 MΩ) versus human motion time

The proposed S-TENG has been used for the sensing of human perspiration. As shown in Fig. 6a, the S-TENG is connected to the human elbow after different durations of body motion. Afterward, the exposed layer of the human skin is cleaned with a dry towel and elbow motion ensues (Fig. 6; the collected electronic output is shown in Fig. 6c). The trend observed is similar to that shown in Fig. 5, i.e., from the viewpoint of the correlation between electronic output (conforming load: 100 MΩ) and the duration of human motion. The results indicate that the proposed S-TENG can be employed for sensing human perspiration and monitoring human motion time.

**Fig. 7:**
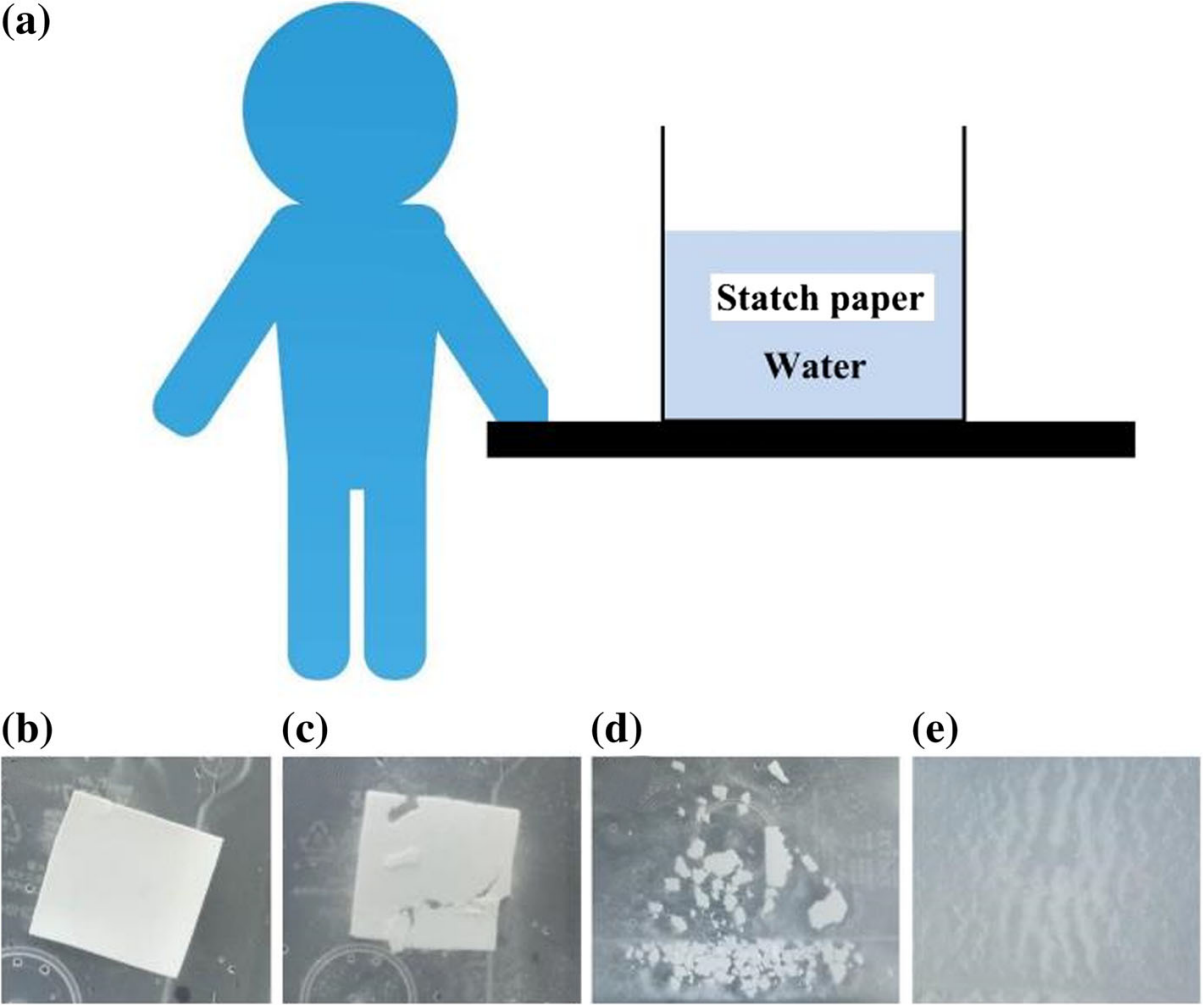
**a** Degradability tests performed by dipping starch paper in water, **b** immediately, and after **b** 1, **c** 2, **d** 3, and **e** 4 min

The disposability attributes of the starch paper were determined by evaluating the dissolution activity, as shown in Fig. 7. During this determination, the paper was immersed in tap water under gentle vibration by human hand, as depicted in Fig. 7a, for various durations (see Fig. 7b–e). The starch paper broke down completely within 4 min, indicating that the proposed S-TENG may be fully degradable.

## Conclusion

In this work, a new and simple methodology for fabricating disposable TENG devices employing ecofriendly biodegradable starch papers is introduced. The rapid and simple process for constructing the S-TENG utilizes cost-effective and commercially accessible materials. The starch structure can be broken down into powder in water within 4 min. The proposed TENG has considerable potential in the field of wearable electronics.

## Abbreviation

TENG: Triboelectric nanogenerator
